# Influence of Food Matrices on the Stability and Bioavailability of Abrin

**DOI:** 10.3390/toxins10120502

**Published:** 2018-12-01

**Authors:** Christina C. Tam, Thomas D. Henderson, Larry H. Stanker, Luisa W. Cheng

**Affiliations:** Foodborne Toxin Detection and Prevention Research Unit, Western Regional Research Center, Agricultural Research Services, United States Department of Agriculture, 800 Buchanan Street, Albany, CA 94710, USA; christina.tam@ars.usda.gov (C.C.T.); thomas.henderson@ars.usda.gov (T.D.H.II); larry.stanker@ars.usda.gov (L.H.S.)

**Keywords:** Abrin, *Abrus precatorius*, mouse bioassay, food safety, thermal inactivation, food matrices, milk, eggs, ground beef, pasteurization

## Abstract

Abrin, a highly toxic plant toxin, is a potential bioterror weapon. Work from our laboratory and others have shown that abrin is highly resistant to both thermal and pH inactivation methods. We sought to evaluate the effectiveness of selected food processing thermal inactivation conditions against abrin in economically important food matrices (whole milk, non-fat milk, liquid egg, and ground beef). The effectiveness of toxin inactivation was measured via three different assays: (1) In vitro cell free translation (CFT) assay, (2) Vero cell culture cytotoxicity; and the in vivo mouse intraperitoneal (ip) bioassay. For both whole and non-fat milk, complete inactivation was achieved at temperatures of ≥80 °C for 3 min or 134 °C for 60 s, which were higher than the normal vat/batch pasteurization or the high temperature short time pasteurization (HTST). Toxin inactivation in liquid egg required temperatures of ≥74 °C for 3 min higher than suggested temperatures for scrambled eggs (22% solids) and plain whole egg. Additionally, the ground beef (80:20%) matrix was found to be inhibitory for full toxin activity in the mouse bioassay while retaining some activity in both the cell free translation assay and Vero cell culture cytotoxicity assay.

## 1. Introduction

Members of the Type II family of ribosome-inactivating proteins (RIP), such as abrin and ricin, inhibit eukaryotic protein synthesis, thereby leading to apoptosis and cell death. Abrin is an A-B toxin with the A-chain having an *N*-glycosidase activity that cleaves the C–N bond of adenine at position 4324 on the 28S rat ribosomal RNA [1,2]. This cleavage prevents ribosomes from binding to elongation factors (EF) 1/2, which leads to eventual cell death [1,2,3,4,5,6,7]. The B-chain is important for binding to cell surface carbohydrate receptors facilitating receptor-mediated endocytosis of the toxin.

Abrin intoxication can occur via three routes: Cutaneous, inhalational, and gastrointestinal. Ingestion of *Abrus precatorius* seeds, usually by children, has no detrimental effects if the hard shell of the seed is not disrupted [8]. Supportive care is the primary treatment given for abrin intoxication due to the lack of an antidote. Oral intoxication with abrin is treated with emesis induction, gastric lavage, activated charcoal, and whole bowel irrigation [5].

In humans, the oral LD_50_ is approximately 10 to 1000 μg/kg and 3.3 μg/kg if injected [9]. In mice, abrin is more lethal than ricin with an intravenous LD_50_ of 0.7 μg/kg vs. 22 μg/kg [10]. Abrin poisoning has usually been due to the accidental ingestion of hard rosary pea seeds used in traditional jewelry [11]. Oral ingestion of the hard rosary pea seeds rarely cause death as long as the hard seed shells are intact. However, due to abrin’s extreme toxicity and the ease in obtaining the seed, as well as the simple purification process, there is significant concern regarding the deliberate use of this toxin by bioterrorists [12,13]. In a case of the abrin suicide attempt, the patient ordered rosary pea seeds online from Asia and ingested approximately 10 seeds in his suicide attempt, but luckily did not die because supportive care was given early [13]. If the ingestion of 10 seeds was sufficient to cause illness, one can imagine the morbidity and mortality effects that would ensue if our food supply were ever targeted by bioterrorists using purified/and or crude abrin. Deliberate contamination of the food supply would most likely occur at the food processing and/or manufacturing facilities with the toxin itself instead of seeds, which would present as a gastrointestinal illness at our hospitals and clinics. Even though intoxication via oral ingestion is less efficient than with other possible routes, the LD_50_ for oral ingestion is 10 to 1000 μg/kg, which means that the contamination with abrin at microgram/mL or milligram/mL in food and beverages is certainly more than sufficient to cause 50% death due to consumption of more than 1 mL or 1 gram of liquid or solid food. Even contamination with abrin at lower levels can cause illness and overwhelm our public health response. To ensure our nation’s food safety, a rigorous examination of current food safety processing practices and their effect on the stability and bioavailability of abrin in various food matrices is needed. The knowledge gained from these studies will help us to mitigate the effects of the deliberate weaponization of abrin and maybe other highly toxic RIPs, like ricin, by bioterrorists.

Only limited data is available on the effect of common food processing inactivation procedures on abrin. Previous studies suggest that abrin is extremely heat stable and pH tolerant [14,15,16]. Jackson et al. [16] found that food matrices, such as dairy, negatively affected the biological activity of the abrin and toxin inactivation in dairy required exposure to 85 °C for 30 min as assayed by an enzyme-linked immunosorbent assay (ELISA). In our previous study on the stability and bioavailability of abrin in phosphate gelatin buffer, we determined that abrin needed to be exposed to temperatures ≥74 °C for 3 min to completely inactivate toxin activity, whereas pH had no effect [17]. In this study, we will determine the influence of four different food matrices (whole milk, non-fat milk, liquid egg, and ground beef) on the stability and bioavailability of abrin after exposure to some currently suggested minimal food processing procedures using thermal heat as the inactivation parameter. The study will use three different assays to fully assess the effects of thermal inactivation on abrin A- and B-chain: (1) Measuring active abrin A-chain activity via an in vitro cell free translation assay (catalytic inactivation resulting in protein synthesis); (2) an in vitro cell culture Vero cell cytotoxicity assay (measuring both A- and B-chain functions); and (3) an in vivo validation with the mouse ip bioassay to determine the activity of the whole toxin (A- and B-chain) systemically due to the difference in results that have occurred using each of the assays [17].

## 2. Results

### 2.1. Abrin Inactivation in Whole Milk and Non-Fat Milk Require Similar Conditions

We sought to evaluate the effectiveness of some common pasteurization conditions for dairy and their effect on the highly potent plant toxin, abrin. From the International Dairy Foods Association, three conditions were chosen to be tested: (1) Vat/batch pasteurization [63 °C (145 °F) for 30 min], (2) high temperature short time pasteurization [HTST—72 °C (161 °F) for 15 s], and (3) ultra-pasteurization [138 °C (280 °F) for 2 s]; however, we did not have the equipment that would reach the ultra-pasteurization temperatures. Therefore, to mimic ultra-pasteurization, we inactivated the toxin at 134 °C (273.2 °F) for 2 s, 30 s, or 60 s using a heat-block. In addition to these conditions, we also inactivated the toxin using the conditions that we showed previously to completely inactivate abrin activity in phosphate gelatin buffer [17].

**Figure 1:**
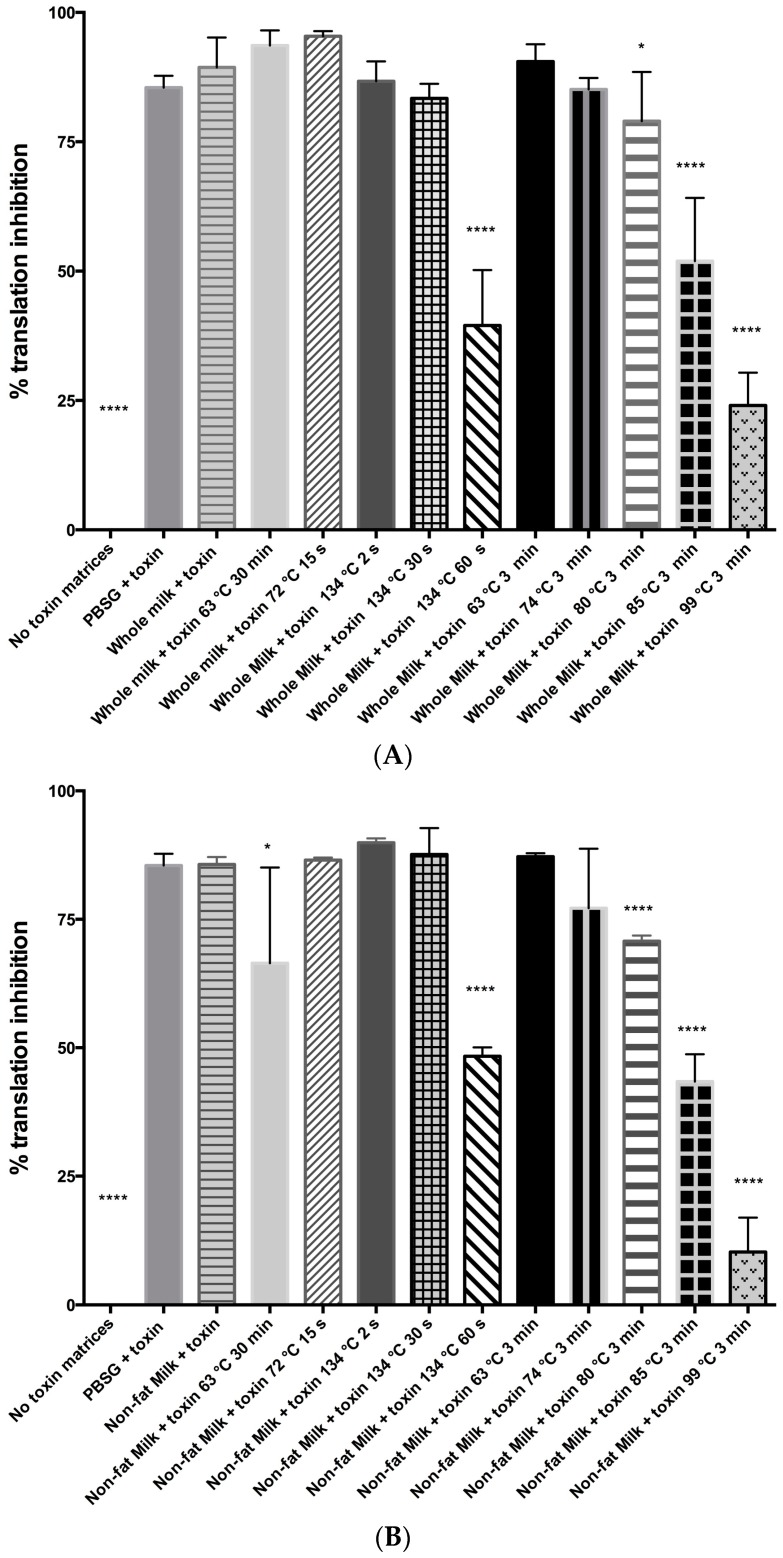
High temperatures and increased exposure times were required to reduce abrin’s translation inhibition activity in milk matrices. (**A**) 100 ng/mL of toxin from each condition was tested in the cell free translation assay. Abrin translation inhibition activity in whole milk was reduced as temperatures and exposure times were increased. (**B**) Reduction of abrin’s translational inhibitory activity in non-fat milk was observed at higher temperatures and increased exposure times. Phosphate gelatin (PBSG) with or without toxin was added as controls for the assay. Whole milk and non-fat milk were treated with or without toxin and then either subjected or not to the inactivation conditions. The graphs represent the cumulative data from two independent experiments with *n*= 6 wells in total. Means of six samples ± standard deviation (SD) are shown. A two-tailed unpaired Student’s *t*-test was used for determining statistical significance, (****) *p* < 0.0001; (*) *p* < 0.05.

In Figure 1A, we showed the results of abrin inactivation in whole milk as measured by the enzymatic activity of the A-chain. Vat/batch pasteurization as well as high temperature short time conditions were not able to reduce the inhibition seen in protein synthesis due to abrin A-chain activity. Inactivation at 80 °C for 3 min in whole milk caused a statistically significant decrease in translational inhibition as compared to abrin in whole milk (79% to 90%, *p* = 0.045). However, the most significant decrease in translation inhibition activity were seen after abrin inactivation at ≥85 °C for 3 min or 134 °C 60 s (*p* < 0.0001). Figure 1B shows a very similar profile for abrin inactivation and reduction in A-chain activity in non-fat milk compared to whole milk. Vat/batch pasteurization at 63 °C for 30 min had a significant reduction in translation inhibition compared to abrin in non-fat milk (66% to 86%, *p* = 0.03) with the caveat that the standard deviations (SD) are large. Regardless, these results indicate that abrin was still active in the commonly used vat/batch pasteurization as well as the high temperature short time pasteurization method, and that higher temperatures and exposure times are needed to thermally inactivate abrin.

Since abrin in both whole milk and non-fat milk retained catalytic activity in the protein translation assay following treatment with the vat/batch and HTST conditions, we wanted to know if any of the inactivation treatments would affect abrin activity in the in vitro Vero cell cytotoxicity assay, since this assay measures both toxin binding/internalization (B-chain) and catalytic activity (A-chain). Table 1 summarizes the results of these studies. The conditions that reduced the cell cytotoxicity of abrin intoxication were inactivation at temperatures ≥80 °C for 3 min or 134 °C for 60 s (*p* < 0.0001).

**Figure 2:**
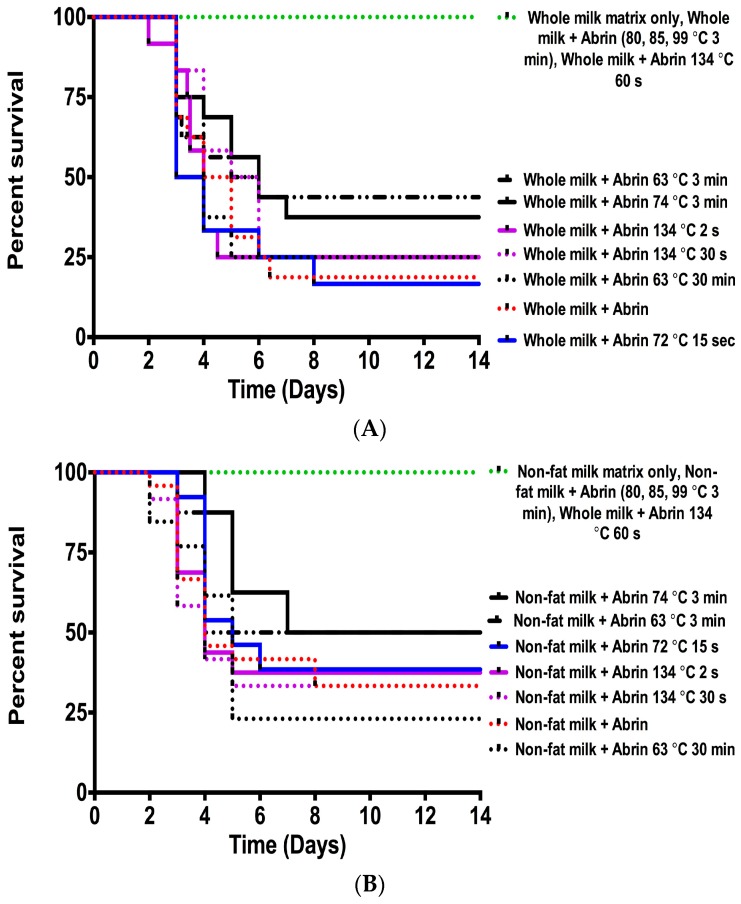
Exposure to high temperatures is required for full toxin inactivation and survival in both whole milk and non-fat milk matrices. (**A**) Mice administered by intraperitoneal injection (ip) with toxins treated at temperatures ≥ 80 °C for 3 min or 134 °C for 60 s in whole milk survive abrin intoxication. (**B**) Mice survive abrin intoxication in non-fat milk only if they have been inactivated at ≥ 80 °C for 3 min or 134 °C for 60 s. Mice were given non-treated or various heat-treated abrin at a lethal dose of 1 μg per mouse by ip (*n* = 4 mice per experimental condition) for two to four independent experiments with *n* = 8–16 mice per cohort in total. Cumulative survival curves were plotted for each condition using GraphPad Prism 6. A statistically significant decrease (*p* < 0.0001) in toxicity was seen in abrin treated at temperatures ≥80 °C for 3 min (green dotted line) or 134 °C for 60 s (green dotted line) compared to abrin non-treated in either whole milk (red dotted line in **A**) or non-fat milk (red dotted line in **B**) using the log-rank (Mantel-Cox) test.

The cell cytotoxicity data suggested that only those toxins treated at ≥80 °C for 3 min or at 134 °C for 60 s will be unable to cause death in the mouse bioassay. To confirm this hypothesis, mice were administered by intraperitoneal injections (ip) abrin toxin with or without heat inactivation at 1 µg per mouse and survival was monitored. In Figure 2A, the survival of mice given abrin in whole milk without inactivation (red dotted line) is shown. This cohort had an 18% survival rate with a median survival of 4.5 days. Mice dosed with matrix alone, with abrin in whole milk treated at ≥80 °C for 3 min, and with abrin in whole milk treated at 134 °C 60 s had 100% survival at 14 days (green dotted line), *p* < 0.0001. As shown in Figure 2A, mice given toxin inactivated with various other conditions were still lethal (all lines except for red dotted) in the mouse bioassay in comparison to the conditions that gave 100% survival (green dotted line). There are slight variations in both survival percentages and median survival time, but these differences were not statistically significant when compared against the non-heat inactivated toxin in whole milk. Since the survival curves represent the survival of each mouse for every independent experiment, we can expect some potential inter-and intra- assay variation.

Figure 2B shows a similar survival curve for mice dosed with abrin in non-fat milk. Mice given abrin in non-fat milk without inactivation had a 33% survival rate with a median survival of four days (red dotted line). The only cohorts that survived in the non-fat matrix study were the matrix alone, abrin treated at ≥ 80 °C for 3 min, and abrin at 134 °C for 60 s (*p* < 0.0001). As seen with mice given toxin at all the other inactivation conditions in whole milk, these conditions were also not effective in the non-fat milk matrix due to the significant mortality seen in the mouse bioassay. We also can see that there are both non-statistically significant variations with both survival percentages and median survival time in the non-fat milk survival assay that is most likely due to inter-and intra-assay variability. Of note, there is no statistically significant difference between the whole milk vs. non-fat milk survival curves when comparing the same experimental condition in both matrices.

### 2.2. Minimal Processing Temperatures and Times for Plain Whole Egg and Scrambled Eggs (22% Solids) Are Insufficient for Inactivation of Abrin

**Figure 3:**
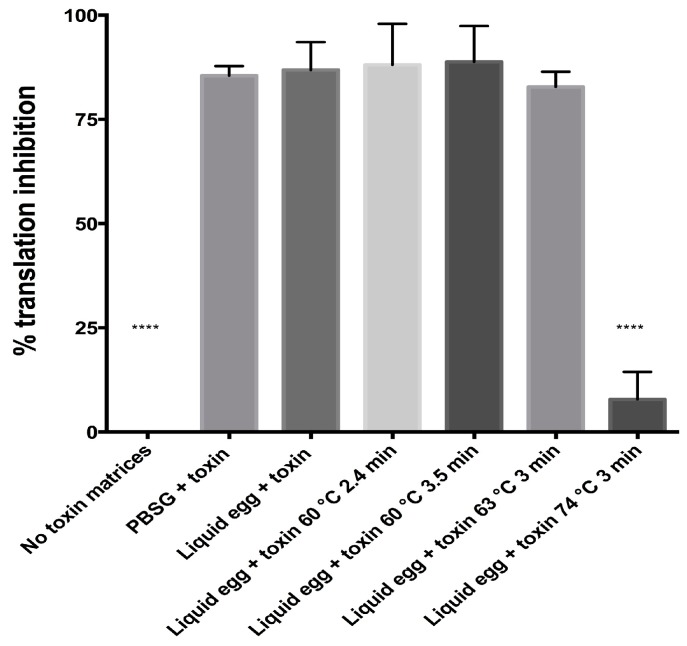
Abrin in liquid egg matrix (egg yolk and egg white) exposed to plain whole egg and scrambled egg inactivation parameters retain full translational inhibition activity. Abrin translation inhibition activity in liquid egg was not reduced using either the plain whole egg (60 °C for 3.5 min) or scrambled egg (22% solids) (60 °C for 2.4 min) inactivation conditions. Reduction of abrin’s translational inhibitory activity in liquid egg was sufficient at ≥ 74 °C for 3 min with the caveat that the liquid egg “cooks” and forms solids. Phosphate gelatin (PBSG) with or without toxin was added as controls for the assay. Liquid egg was treated with or without abrin and then either subjected or not to the inactivation conditions. The graphs represent the cumulative data (means of six samples ± standard deviation (SD) from two independent experiments. A two-tailed unpaired Student’s *t*-test was used to determine statistical significance, (****) *p* < 0.0001.

Abrin inactivation in liquid egg (egg yolk and egg white) was tested using two conditions from the International Egg Pasteurization Manual: (1) 60 °C for 3 min was the temperature chosen for pasteurization of plain whole egg (egg yolk and egg white) and (2) scrambled eggs (22% solids) were pasteurized at 60 °C for 2.4 min. Additionally, we subjected the liquid egg to temperatures ranging from 63 °C to 99 °C for 3 min (same conditions for milk matrices). We found that both the plain egg and scrambled egg pasteurization conditions did not inactivate abrin toxin A-chain activity in the cell-free translation assay in the liquid egg matrix (Figure 3). Inactivation at 74 °C for 3 min dramatically reduced toxin activity in the cell-free translation assay compared to abrin in liquid egg (8% to 87% respectively, *p* < 0.0001). However, as we increased the inactivation parameters from 63 °C to 74 °C for 3 min, we found that our viscous liquid egg mixture ‘cooked’ and started forming semi-solid to more solid structures above 74 °C. We resuspended the solid structures to the best of our ability, spun down the mixture, and obtained the supernatants for all further procedures. Therefore, we have to caveat these results because we cannot say with certainty that the toxin was not sequestered into the solids and potentially titering away the toxin activity. However, we clearly demonstrated that the plain egg and scrambled egg pasteurizations conditions were not sufficient to inactivate abrin A-chain catalytic activity for abrin in the liquid egg matrix.

Table 2 summarizes the results of the cell cytotoxicity assay with the liquid egg toxin samples. As seen with the translation inhibition assay, only inactivation at ≥ 74 °C abrogated the cytotoxicity associated with intoxication with abrin (*p* < 0.0001). However, the same caveat holds with the potential titering away of toxin in the solids that form at inactivation temperatures of ≥ 74 °C.

**Figure 4:**
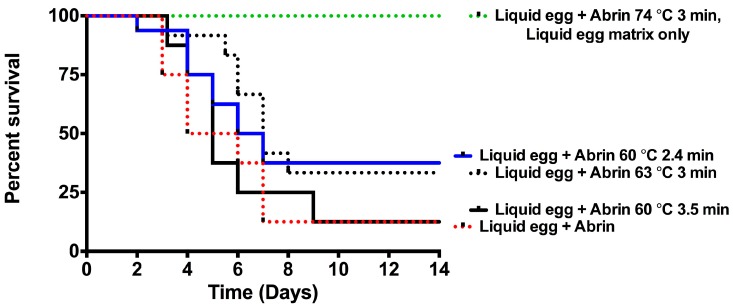
Complete abrin inactivation and mice survival required abrin in liquid egg to be exposed to temperatures ≥ 74 °C for 3 min. Mice administered intraperitoneal injection (ip) with abrin treated at ≥74 °C for 3 min in liquid egg survived abrin intoxication. Mice were given non-treated or heat-treated abrin: Liquid egg samples via ip at 1 μg per mouse (*n* = 4 mice per experimental condition) for two to four independent experiments with *n* = 8–16. Survival curves were plotted for each condition using GraphPad, Prism 6. A statistical significant decrease (*p* < 0.0001) in toxicity was seen in abrin treated at ≥74 °C for 3 min (green dotted line) compared to non-treated abrin in liquid egg (red dotted line) from the log-rank (Mantel-Cox test).

The in vivo mouse bioassay was used to evaluate the effects of abrin intoxication in liquid egg mixes subjected or not to heat-inactivation. Figure 4 shows that mice given abrin liquid egg mixtures exposed to temperatures ≥74 °C for 3 min (green dotted line) all survived to the end of the experiment compared to mice given abrin liquid egg (red dotted line) (*p* < 0.0001). Whether the mice survived because of the titration of toxin in the solids or that it was due to affecting the A and/or B-chain of abrin is currently unknown.

### 2.3. Ground Beef Affects the Bioavailability of Abrin

**Figure 5:**
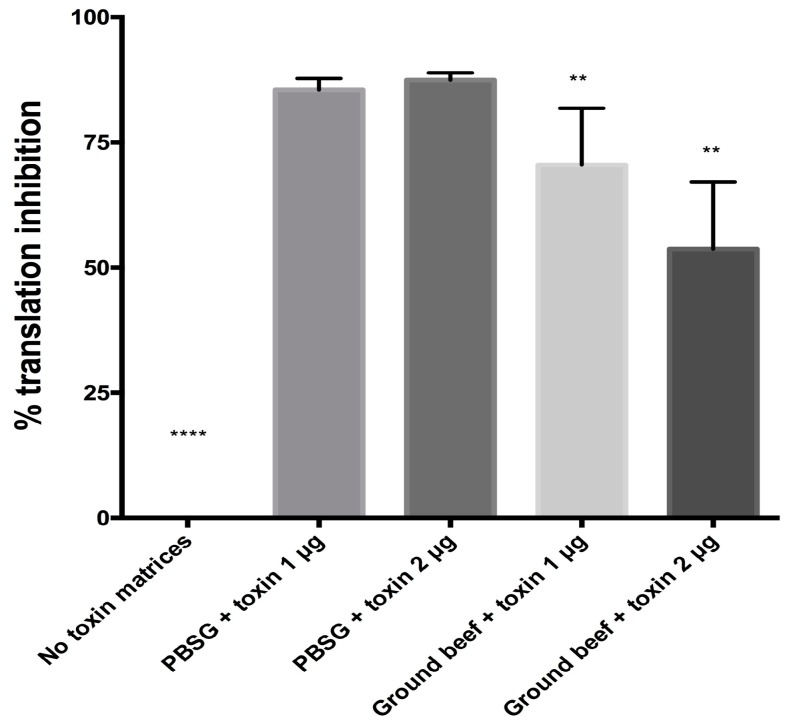
Abrin spiked into ground beef has reduced A-chain enzymatic activity. Phosphate gelatin (PBSG) with or without toxin was added as controls for the experiments. Ground beef spiked with two different concentrations of abrin were tested for inhibition of protein synthesis. The graphs represent the cumulative data [means of six samples ± standard deviation (SD) from two independent experiments with *n* = 6 wells in total. A two-tailed unpaired Student’s *t*-test was used for statistical significance, (****) *p* < 0.0001; (**) *p* < 0.05.

The effect of the ground beef matrix on toxin stability and bioavailability was assessed. We found that there was a significant reduction in abrin activity (the inhibition of protein synthesis in the in vitro translation assay) in ground beef as compared to abrin spiked into phosphate gelatin (PBSG) (Figure 5). An 85% translation inhibition was observed when 1 µg of abrin in PBSG was tested. Samples of ground beef + abrin at the same dose resulted in a 70% inhibition, *p* = 0.01. An even greater inactivation of abrin was observed with the 2 µg dose, phosphate gelatin was able to inhibit 87% while the ground beef mixture only inhibited 54%. This suggests that there may be factors in the ground beef that may be potentially inactivating or the ground beef is sequestering the toxin.

Next, the ground beef toxin mixtures were tested in the cell cytotoxicity model. As observed above using the cell-free translation assay, there were significant reductions in toxin cytotoxicity in both ground beef toxin samples compared to their controls in phosphate gelatin (PBSG) (*p* < 0.0001) (Table 3).

**Figure 6:**
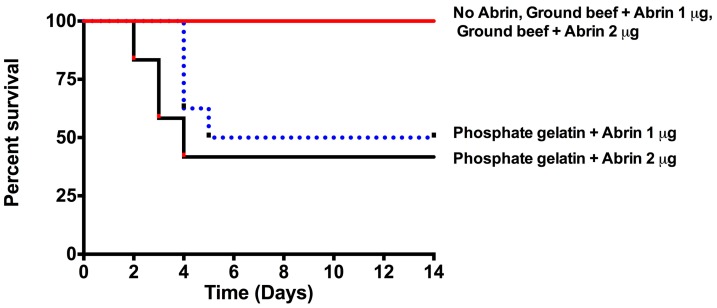
Ground beef matrix inhibits abrin intoxication in the mouse bioassay. Mice administered toxin in ground beef completely survive abrin intoxication. Abrin in ground beef was given to mice at a lethal dose of 1 μg or 2 μg per mouse (*n* = 4 mice per experimental condition) for two to three independent experiments with a total of *n* = 8–12 mice per cohort in total. Cumulative survival curves were plotted and a statistically significant decrease in toxicity was seen in abrin spiked into ground beef compared to abrin present in phosphate gelatin (*p* < 0.05) (log-rank/Mantel-Cox test).

As shown in Figure 6, all the mice injected with abrin spiked ground beef supernatants survived (red line). In contrast, only 41% (phosphate gelatin + Abrin 2 µg) or 50% (phosphate gelatin + Abrin 1 µg) of the mice injected in buffer survived (black line and blue dotted line) (*p* < 0.05). These results confirm the detrimental effect of the ground beef matrix on toxin bioavailability previously in the two in vitro assays.

## 3. Discussion

In this study, we determined the influence of food matrices on the stability and bioavailability of abrin, an extremely potent plant toxin that is of a food safety and bioterror concern. Previous studies have indicated that some commonly used pasteurization methods for milk were not sufficient for abrin toxin inactivation [16,18]. Our studies with both whole and non-fat milk support these claims. Both the vat/batch pasteurization (63 °C, 30 min) and high temperature short time (HTST) (72 °C, 15 s) do not abrogate abrin’s translational inhibitory activity (Figure 1A,B), cell culture cytotoxicity (Table 1), and in vivo mouse survival assay (Figure 2). Toxin mixtures in milk matrices required temperatures of ≥80 °C for 3 min or 134 °C for 60 s to show dramatic reductions in cell culture cytotoxicity (Table 1) that correlated with survival in the mouse bioassay (Figure 2). These results were different than the previous study findings that showed complete inactivation of abrin in milks exposed to 85 °C for 30 min [16], but their study used an ELISA format as determining inactivation whereas we used different assays. Regardless, both studies were correct in stating that higher temperatures with increasing exposure times were necessary to inactivate abrin. Additionally, our previous study concerning abrin inactivation in phosphate gelatin buffer suggested that temperatures of ≥74 °C for 3 min were sufficient [17]. However, this study suggested that both milk matrices may be protective of abrin inactivation and is in agreement with previous studies of abrin as well as ricin [16,19,20].

Inactivation of liquid egg contaminated with abrin using either the plain egg or scrambled egg (22%) pasteurization methods were unsuccessful. Toxins inactivated with these two parameters were still catalytically active in the cell free translation assay (Figure 3), cytotoxic in the Vero cell assay (Table 2), and were lethal to mice in the mouse bioassay (Figure 4). The only successful method to inactive abrin in liquid egg was to expose the mixture to temperatures ≥ 74 °C for 3 min, which almost completely abrogated the translational inhibition activity (Figure 3), had dramatically reduced cell cytotoxicity in the Vero cell assay (Table 2), and was completely abrogated for toxicity in the mouse bioassay (Figure 4). However, these results have a caveat to them. Increasing the temperature from 63 °C to 74 °C forms a semi-solid structure (liquid egg mixture is cooked) while further increases resulted in the formation of more ‘solid’ structures. Since the assays were from the cleared supernatants from these samples, there is the possibility that toxin was titered away into the solid portion and hence why one sees abrogation of the abrin phenotype.

We have found that ground beef (80:20%) affected the bioavailability of abrin substantially. Toxin spiked into ground beef had reduced translational inhibitory activity (Figure 5) in comparison to toxin spiked into phosphate gelatin buffer. This led to at least a two-fold decrease in Vero cell cytotoxicity (Table 3) and complete survival in the mouse bioassay (Figure 6). There seems to be a component in the ground beef mixture, i.e., potentially fat, that could be binding to and sequestering abrin from the aqueous phase after centrifugation to generate cleared lysates for the three subsequent assays. The effect could be that the ‘effective dose’ of abrin that we are testing is at least two-fold reduced and hence resulting in low toxicity.

We have shown in this study that food matrices significantly affect the stability and bioavailability of abrin. Since abrin is a member of the Type II family of ribosome-inactivating proteins (RIP), the question that arises is how relevant are these results to other RIPs, such as ricin or ebulin [21,22,23,24,25,26]? The Type II family of ribosome-inactivating proteins has been further delineated into toxic and non-toxic families dependent on their ability to cause mammalian cytotoxicity. Both abrin and ricin are members of the toxic subfamily whereas ebulin and nigrin b are considered non-toxic. These RIPs and related proteins have in vitro translation inhibition activity (may be dependent on the presence of reductant for maximal activity). However, both ebulin (and its various isoforms) as well as nigrin b have significantly reduced cellular cytotoxicity even though they both have B-chains [22,23,24,25,26].

Abrin is more toxic than ricin, which itself is more toxic than ebulin or nigrin b on the order of log differentials. In mice, the iv dose for abrin is 0.7 μg/kg whereas ricin is 22 μg/kg when given intravenously [10]. Recently, ricin has been shown to have an LD_50_ of 29 mg/kg (ig), 0.023 mg/kg (ip), and 0.008 mg/kg (iv) [27]. Ebulin 1 has an LD_50_ of 2.5 mg/kg (ip) and ebulin f has an oral LD_50_ of 5 mg/kg and 2.8 mg/kg (ip) [22,24,25,26,28]. The differences in cytotoxicity amongst abrin, ricin, ebulin, and nigrin b may be due to the following factors: (1) The differences in B-chain specificity, i.e., binding to galactosyl receptors that are more abundant vs. other receptors, including sialic acid moieties; (2) the route of internalization used by the various toxins, i.e., endoplasmic reticulum (ER)/retrograde transport (ricin/abrin) vs. other routes; and (3) the ability to avoid/inhibit degradation in the host cell, i.e., unfolded protein response (UPR), ricin is able to block this response [29,30], but the non-toxic type II RIPs may not. Therefore, the effects of food matrices on these various toxins may not necessarily be the same. However, ricin inactivation has been shown to require higher temperatures and longer exposure times similar to abrin using whole milk, skim milk, and infant milk [16,31].

We have found that abrin is highly resistant to thermal inactivation procedures in whole milk, non-fat milk, and liquid egg. Selected common current food processing procedures were unable to completely inactivate abrin toxin similar to results reported earlier for ricin [19,20] Additionally, we found that ground beef reduced the bioavailability of abrin by most likely reducing the ‘effective’ dose given to animals and thus may affect potential detection assays using this matrix. This study supports and expands on previous research from different groups that leads to the idea that some current food processing conditions may need to be adjusted to protect the food supply from protein contaminants, like abrin and ricin.

## 4. Materials and Methods

### 4.1. Materials

Abrin toxin consisting of mixed isomers at 5 mg of lyophilized powder (Cat# ABR-1) was bought from Toxin Technology, Inc. (Sarasota, FL, USA). Abrin toxin was resuspended with 1 mL of 1× phosphate buffered saline (PBS) pH 7.2 to give a 5 mg/mL stock stored at 4 °C. The following items were purchased from Promega (Madison, WI, USA): Nuclease-treated rabbit reticulocyte lysate (L4960), complete amino acid mixture (1 mM, N2111), nuclease-free water (P1193), luciferase mRNA (1 mg/mL, L4561), and the Bright-Glo Luciferase Assay System (10 mL, E2610). Whole milk, non-fat milk, ground beef (80:20%), and eggs were obtained from a local supermarket.

### 4.2. Bioavailability of Abrin in Whole Milk and Non-Fat Milk after Pasteurization Conditions

Abrin was diluted into 100 μL of various matrices (1× PBS pH 7.2 + 0.2% phosphate buffer gelatin, whole milk, non-fat milk) in a PCR tube and incubated for 30 min at room temperature. A thermocycler was used to heat-inactivate the toxins. The temperatures were associated with the minimal temperatures and times as suggested by the International Dairy Foods Association (IDFA) for pasteurization as well as from previous stability work [17]. After temperature treatment, the toxin doses were diluted with 900 μL of 1× PBS pH 7.2 to yield either 10 or 20 μg/mL. Samples per condition were split for the mouse bioassay, cell free translation, and the Vero cell culture assay. Abrin at 100 ng/mL was used for the cell free translation assay while 5 ng/mL was used for the Vero cell assay. The two-tailed unpaired Student’s *t*-test was used to evaluate statistical significance between abrin in matrix not exposed to heat vs. the various heat-treated abrin: Matrix samples. *n* = 4 mice per experimental condition were intoxicated via ip at a dose of 1 μg per mouse. GraphPad Prism 6 (GraphPad Software, La Jolla, CA, USA) was used to plot survival curves for each condition per independent experiment. Final survival curves were obtained from all independent experiments. *p*-values < 0.05 were considered significant using the log-rank (Mantel-Cox) test.

### 4.3. Bioavailaibity and Thermal Stability of Abrin Toxin in Ground Beef

Ground beef (80:20%) was added to PCR tubes at 0.1 g per 100 μL of 1× PBS pH 7.2 + 0.2% phosphate buffer gelatin and incubated at room temperature for 30 min. The toxin doses were diluted with 900 μL of 1× PBS pH 7.2 to yield either 10 or 20 μg/mL. Each tube was vortexed vigorously for 1 min and centrifuged to remove particulates at 10,000× *g* for 5 min [17,32]. Toxin samples per condition were split for the mouse bioassay, cell free translation, and the Vero cell culture assay. The cell free translation assay used abrin at 100 ng/mL whereas the Vero cell cytotoxicity assay was used at 5 ng/mL. Statistical significance between the non-heat-treated abrin sample with each of the other heat-treated toxin samples was calculated using the two-tailed unpaired Student’s *t*-test. Mice were administered 1 μg or 2 μg per mouse via ip with *n* = 4 mice per experimental condition. Cumulative survival curves were generated for each condition and *p*-values < 0.05 were considered significant by the log-rank (Mantel-Cox) test.

### 4.4. Abrin Stability and Toxicity in Liquid Egg Exposed to Thermal Heat

100 μL of freshly cracked and mixed liquid egg (yolk and egg white) was placed in PCR tubes. Toxin was added and the toxin: matrix tubes were incubated for 30 min at room temperature. A thermocycler (Eppendorf, Hauppauge, NY, USA) was then used to inactivate the toxin present in the matrix. Temperatures chosen were suggested by the International Egg Pasteurization Manual and our previous study [17]. After temperature treatment, the toxin doses were diluted with 900 μL of 1× PBS pH 7.2 to yield either 10 or 20 μg/mL. Toxin samples per condition were split for the mouse bioassay, cell free translation, and the Vero cell culture assay. Cell free translation assay was performed using 100 ng/mL while 5 ng/mL was used for the Vero cell assay. Statistical significance between the abrin: liquid egg sample with each of the other heat-treated toxin: matrix samples was assessed using the two-tailed unpaired Student’s *t*-test. Mice were given 1 μg per mouse via ip with *n* = 4 mice per experimental condition. Survivals of mice for each condition from all independent experiments were pooled and final survival curves were graphed using GraphPad Prism 6. The log-rank (Mantel–Cox) test determined statistical significance between the survival curves with *p*-values < 0.05 considered significant.

### 4.5. Abrin In Vitro Cell Free Translation Assay

The in vitro cell free translation assay has been described previously [20,32]. Briefly, toxin: matrix samples were diluted to 100 ng/mL with 1× PBS pH 7.2. A translational lysate mixture consisting of the following at a ratio of (*v*/*v*) [35:1:1:36:2]: Nuclease-treated rabbit reticulocyte lysate, complete amino acid mixture, RNasin, nuclease-free water, and luciferase mRNA was made. These diluted toxin samples were added to this mixture at a ratio of toxin to translational lysate mixture of 1:5 (3 μL to 15 μL). After gentle mixing, we concentrated the entire reaction mixture via a quick spin and incubated at 30 °C at 80 rpm for 90 min. Each reaction mixture was added in triplicate to wells containing 5 μL of the reaction in a 96-well black plate. 100 μL of the Bright-Glo Luciferase Assay System was then added and luminescence was measured on a Victor 3 (Perkin-Elmer, Shelton, CT, USA). The full translation efficiency was averaged using the negative control buffer as well as the negative controls consisting of the various matrix dilutions. Toxin activity was calculated as percentage (%) of translation inhibition due to inhibition of translation [(negative control cps—toxin sample cps)/negative control cps] × 100. All values shown represent the mean ± standard deviation (SD) of triplicate samples measured in a representative experiment. Statistical significance was calculated using the two-tailed unpaired Student’s *t*-test from GraphPad Prism 6 with *p*-values < 0.05 considered significant.

### 4.6. Vero Cell Cytotoxicity Assay

Vero cells were grown in Dulbecco’s Modified Eagle Medium high glucose + 10% fetal bovine serum in a humidified incubator (37 °C, 5% CO_2_). Cells were seeded in 5 × 10^3^ cells/100 uL into black-sided, clear-bottom 96-well tissue culture plates overnight (18 hours) at 37 °C. The old media was removed and 100 μL of fresh DMEM—containing DMEM only, untreated abrin matrices, or the toxin: matrix complexes inactivated at different temperatures—was then added. After incubation for 2 h at 37 °C, the media was removed and more fresh media was added. Cell were incubated for 48 h at 37 °C before measuring luminescence. CellTiter-Glo (Promega, G7570) was diluted 1:5 in PBS and 100 μL was added to each well. The plate was shaken for 2 min in order to lyse the cells. After incubation for another 10-min at room temperature, luminescence was measured (Victor 3) on a plate reader (lid was removed from the plate for a better signal) [17]. The percentage (%) of cytotoxicity for each well of the toxin treated samples was calculated as follows: [(average DMEM negative control cps—experimental cps)/average DMEM negative control cps] × 100. The average % of cytotoxicity was calculated for all the conditions. The non-treated abrin/abrin mixes were set as 100% relative cytotoxicity. To obtain the relative cytotoxicity percentages (%) for all the other conditions, we calculated [average % cytotoxicity condition/average % cytotoxicity of positive control non-treated abrin] × 100.

### 4.7. Abrin Intraperitoneal Route (ip) Mouse Bioassay

Randomly assigned female Swiss-Webster mice in groups of four mice were challenged by intraperitoneal injection (ip) with abrin toxin resuspended in 1× PBS with 0.2% phosphate buffer gelatin or matrix-toxin stocks at 1 μg or 2 μg per mouse dependent on experiment. Two to four independent experiments were performed. This strain has been determined to be suitable for studies with ricin and abrin [33,34]. *n* = 4 mice were tested per condition for all independent experiments. Animals were monitored for at least 10 days for signs of intoxication or death. All procedures involving animals were reviewed and approved by the Institutional Animal Care and Use Committee of the United States Department of Agriculture, Western Regional Research Center. Animal use protocols for ricin and abrin mouse bioassays (Protocol # 16-2) were approved by the Western Regional Research Center Institutional Animal Care and Use Committee (WRRC- IACUC) on 2 September 2016.

## Figures and Tables

**Table 1:** Vero cell cytotoxicity of abrin in whole milk and non-fat milk matrices.

Treatment	Relative Cytotoxicity (%)
DMEM/Matrices	0
Abrin Whole Milk	97 ± 3
Abrin Whole Milk 63 °C 30 min	98 ± 2
Abrin Whole Milk 72 °C 15 s	97 ± 3
Abrin Whole Milk 134 °C 2 s	89 ± 11
Abrin Whole Milk 134 °C 30 s	93 ± 2
Abrin Whole Milk 134 °C 60 s	22 ± 8
Abrin Whole Milk 63 °C 3 min	93 ± 5
Abrin Whole Milk 74 °C 3 min	87 ± 6
Abrin Whole Milk 80 °C 3 min	24 ± 12
Abrin Whole Milk 85 °C 3 min	19 ± 6
Abrin Whole Milk 99 °C 3 min	21 ± 8
Abrin Non-fat Milk	94 ± 6
Abrin Non-fat Milk 63 °C 30 min	87 ± 7
Abrin Non-fat Milk 72 °C 15 s	90 ± 6
Abrin Non-fat Milk 134 °C 2 s	95 ± 5
Abrin Non-fat Milk 134 °C 30 s	83 ± 3
Abrin Non-fat Milk 134 °C 60 s	0
Abrin Non-fat Milk 63 °C 3 min	91 ± 5
Abrin Non-fat Milk 74 °C 3 min	74 ± 9
Abrin Non-fat Milk 80 °C 3 min	27 ± 2
Abrin Non-fat Milk 85 °C 3 min	8 ± 3
Abrin Non-fat Milk 99 °C 3 min	6 ± 4

Cytotoxicity of Vero cells treated with matrix/Dulbecco’s Modified Eagle Medium (DMEM), abrin: Matrix, or abrin: Matrix inactivated in various conditions. Cells were treated with toxin at 5 ng/mL. Means of six samples ± standard deviation (SD) from two independent experiments were presented in this table. *p* < 0.0001 for abrin whole milk ≥ 80 °C for 3 min or 134 °C for 60 s as compared to abrin whole milk; abrin non-fat milk ≥ 74 °C for 3 min or 134 °C for 60 s compared to abrin non-fat milk as determined by two-tailed unpaired Student’s *t*-test.

**Table 2:** Cell culture cytotoxicity of abrin toxins in liquid egg.

Treatment	Relative Cytotoxicity (%)
DMEM/Liquid egg	0
Abrin Liquid egg	100 ± 0.1
Abrin Liquid egg 60 °C 2.4 min	96 ± 5
Abrin Liquid egg 60 °C 3.5 min	100
Abrin Liquid egg 63 °C 3 min	87 ± 11
Abrin Liquid egg 74 °C 3 min	23 ± 10

Vero cell cytotoxicity after treatment matrix, Dulbecco’s Modified Eagle Medium (DMEM), abrin in liquid egg, and various heat-treated abrin liquid egg mixes. 5 ng/mL of abrin was used for these assays. Relative cytotoxicity values were calculated from two independent experiments (means of six samples ± standard deviation (SD). The two-tailed unpaired Student’s *t*-test was used to determine statistical significance, *p* < 0.0001 for abrin liquid egg 74 °C for 3 min compared with abrin non-treated.

**Table 3:** The effect of the ground beef matrix on abrin activity in the Vero cell culture cytotoxicity model.

Treatment	Relative Cytotoxicity (%)
DMEM/Matrices	0
Abrin PBSG 1 µg	100
Abrin PBSG 2 µg	100
Abrin Ground beef 1 µg	49 ± 26
Abrin Ground beef 2 µg	39 ± 22

Vero cell cytotoxicity after treatment with matrix, Dulbecco’s Modified Eagle Medium (DMEM), phosphate gelatin buffer (PBSG) toxin, and ground beef toxin mixtures. Cells were treated with 5 ng/mL of toxin for these assays. Means of six samples ± standard deviation (SD) from two independent experiments were used to calculate the relative cytotoxicity. Statistical significance was determined by a two-tailed unpaired Student’s *t*-test, *p* < 0.0001 for all ground beef conditions compared with abrin in PBSG.
